# Brief education and nudging to facilitate TENS use in postoperative care: a quasi-experimental mixed methods study

**DOI:** 10.1186/s12913-026-15913-0

**Published:** 2026-10-27

**Authors:** Salwan Diwan, Paulin Andréll, Axel Wolf, Pether Jildenstål

**Affiliations:** 1 Institute of Health and Care Sciences, University of Gothenburg, Sahlgrenska Academy, Box 457, Gothenburg, 405 30 Sweden; 2 Department of Anaesthesiology and Intensive Care/Pain Centre, Sahlgrenska University Hospital, Region Västra Götaland, Gothenburg, Sweden; 3 Department of Anaesthesiology and Intensive Care, Institute of ClinicalSciences at the Sahlgrenska Academy, University of Gothenburg, Gothenburg, Sweden; 4 Faculty of Health Sciences, Department of Nursing and HealthPromotion Acute and Critical Illness, Oslo Metropolitan University, Oslo, Norway; 5 Department of Health Sciences, Lund University, Lund, Sweden; 6 Department of Anaesthesiology, Surgery and Intensive Care Medicine, Sahlgrenska University Hospital, Gothenburg, Sweden; 7 Faculty of Nursing and Health Sciences, Nord University, Bodø, Norway; 8 Department of Anesthesiology and Intensive Care, Skane University Hospital, Lund, Sweden; 9 Department of Anesthesiology and Intensive Care, Örebro University Hospital and school of Medical Sciences, Örebro, Sweden

**Keywords:** Transcutaneous electrical nerve stimulation (TENS), Nudging, Postoperative pain managment, Implementation strategies, Non-pharmacological interventions

## Abstract

**Background:**

Implementing evidence-based practices into routine clinical care remains a challenge, with traditional implementation strategies yielding modest effects on clinical behavior. In postoperative pain management, evidence-based non-pharmacological modalities such as transcutaneous electrical nerve stimulation (TENS) remain underutilized. Nudging, through subtle modifications to the choice architecture, offers a cost-effective, resource-efficient approach to enhancing adoption and sustaining action at the point of care. This study evaluated whether combining a brief educational workshop with a nudging phase using visual prompts could enhance the adoption of TENS in routine postoperative care.

**Methods:**

An explanatory sequential mixed methods design was applied, combining a quasi-experimental pre–post study with three observation points and qualitative interviews. The study was conducted in a 15-bed postoperative ward at a Swedish university hospital. The intervention comprised a 45-minute hands-on educational workshop followed by a 12-week asynchronous and passive nudging phase. Quantitative data on TENS use were collected from a logbook maintained by healthcare professionals and analyzed with descriptive statistics and comparative tests. To contextualize and explain observed practice changes, semi-structured interviews with a purposive sample of healthcare professionals (*n* = 13) were thematically analyzed.

**Results:**

TENS administrations increased significantly after the interventions compared to baseline. Qualitative findings showed that the interventions reframed TENS from a peripheral option to an integrated component of postoperative pain care. Nudging reinforced awareness, normalized TENS administration in everyday practice, and contributed to early routinization and maintained integration into clinical reasoning.

**Conclusions:**

A brief educational workshop followed by a transparent asynchronous and passive nudging strategy was associated with increased and continued documented use of TENS during the study period, in a setting where no administrations were recorded at baseline despite available devices and a local protocol. This low-resource approach might complement traditional implementation strategies by increasing the visibility of evidence-based non-pharmacological options in routine postoperative care.

**Trial registration:**

ClinicalTrials.gov, NCT06696430. Registered 13 November 2024, https://clinicaltrials.gov/study/NCT06696430.

**Supplementary Information:**

The online version contains supplementary material available at https://doi.org/10.1186/s12913-026-15913-0.

## Contributions to the literature

- This study provides novel evidence on transparent asynchronous and passive nudging as a form of facilitation, illustrating how visual cues can influence practice in postoperative care.
- It demonstrates that a brief, single-session educational workshop, when paired with contextually tailored visual cues, can maintain the adoption of an evidence-based intervention beyond the initial implementation period.
- It shows how transparent asynchronous and passive nudges can function as facilitative mechanisms, reinforcing education and normalizing practice without undermining autonomy.
- It highlights the potential of nudging to enhance the adoption of non-pharmacological pain management strategies, thereby supporting multimodal and guideline-concordant approaches to postoperative care.

## Background

Implementing evidence-based practices into routine clinical care remains a central challenge in healthcare delivery. Despite decades of investment in guideline development and education, only 60% of care aligns with best evidence, while 30% represents low-value care and 10% causes harm [1]. Traditional implementation strategies, such as education and guideline dissemination, are valuable for increasing awareness and supporting professional practice, but systematic reviews suggest that their effects on clinical behavior are often modest, variable, and context dependent [2, 3]. This underscores the need for complementary, cost-effective, and resource-efficient implementation strategies that are seamlessly embedded into existing workflows and capable of enhancing adoption and sustaining action at the point of care.

Nudging refers to subtle modifications to the choice architecture that steer decisions without restricting autonomy or significantly changing economic incentives, and that should be easy and inexpensive to avoid [4]. It has gained international recognition as an innovative and effective approach for promoting decisions that enhance health and well-being with successful applications extending far beyond healthcare, with documented use in financial markets, education policies, and other public sectors [5, 6]. When integrated into routine workflows as low-disruption prompts, nudging can help maintain the salience of evidence-based options and enable sustained implementation over time [7–9]. Within healthcare, nudges have effectively shaped the decision environment, making desired actions more visible, timely, and easy to adopt, and have thereby influenced behaviors ranging from medication prescribing to hand hygiene and adherence to evidence-based guidelines [7, 10–14].

Nudge theory is grounded in the dual-process model of thinking theory, which distinguishes between two modes of thinking: System 1, the fast, automatic, and driven by intuition mode and System 2, the slower, deliberate, and analytical mode [15]. Nudging strategies are designed to target System 1 by subtly guiding behavior without requiring individuals to engage in extensive reasoning for decision-making [16]. Building on the dual-process theory, nudging interventions have also been classified according to two independent dimensions [8, 13]. The first dimension distinguishes between synchronous nudge strategies, delivered at the very moment a decision is being made, and asynchronous strategies, which occur outside of the immediate decision-making situation. The second dimension differentiates active nudges, which require the clinician to engage directly during the decision process, from passive nudges, which influence choices without demanding explicit action at the decision moment.

Postoperative pain management represents a critical area where evidence-based practices are underutilized, despite well-established guidelines promoting multimodal approaches that combine pharmacological and non-pharmacological modalities [17, 18]. Acute postoperative pain remains common, with studies from diverse geographical settings reporting that approximately 40–60% of patients experience moderate to severe pain within the first 24 h after surgery [19–22]. Poorly controlled acute postoperative pain is associated with impaired functional recovery, increased morbidity, higher healthcare utilization, and increased likelihood of developing chronic postsurgical pain (CPSP), commonly defined as pain persisting or recurring beyond 3 months after surgery [19, 23, 24]. A recent review highlights the value of complementary non-pharmacological strategies, such as transcutaneous electrical nerve stimulation (TENS), in reducing pain intensity, lowering opioid consumption, and supporting functional recovery, while minimizing adverse effects [25]. TENS is a low-risk, cost-effective, and a reusable modality that minimizes healthcare waste, supports sustainability, and improves patient comfort and autonomy across various procedures [26–32]. However, TENS adoption in routine postoperative care remains inconsistent, often hindered by low familiarity, workload constraints, limited confidence among healthcare professionals (HCPs), and a prevailing interpretation of multimodal pain managment as primarily pharmacological [33–36].

These barriers position postoperative TENS use as an ideal test case for examining how nudging can act as a facilitator for implementation in multimodal pain management in the postoperative setting. Yet, no studies have tested nudging to enhance adoption of non-pharmacological pain management modalities such as TENS, nor explored the combined effect of brief, hands-on education with targeted behavioral cues to facilitate practices into routine care. This study addresses that gap by evaluating whether a short low-cost educational workshop, followed by a nudging phase using visual prompts, can enhance the implementation and adoption of TENS in postoperative care. The combined approach was designed to initiate and sustain behavior change in real-world conditions, while ensuring feasibility for routine integration.

## Method

### Protocol and registration

The study was registered at ClinicalTrials.gov (NCT06696430) on 13 November 2024, following ethical approval under the Swedish Ethical Review Act (registration number 2024-06961-01). The trial registration also included planned patient interviews, however, these were not conducted due to organizational constraints during the study period. This deviation from the registered protocol is acknowledged for transparency. A related qualitative study from the same clinical context has reported HCPs lived experiences of being nudged to use TENS, focusing on autonomy, ethical acceptability, and professional judgment [37]. The present study differs by focusing on documented TENS use over time across the baseline, post-education, and nudging phases, with qualitative data used to explain and contextualize these quantitative patterns.

### Study design and setting

The intervention was reported in accordance with the Template for Intervention Description and Replication (TIDieR) checklist to ensure transparency and replicability [38]. This study used a single-group quasi-experimental pre–post design with three observation points: baseline (O1), post-education (O2), and post-nudging (O3). The design followed a multiple-intervention format without a control group (O1 × 1; O2 × 2; O3), classified as a Category A quasi-experiment [39]. To complement the quantitative assessment of changes in documented TENS use over time, a qualitative interview component was conducted after the quantitative phase. This component was guided by explanatory sequential mixed methods principles, where qualitative data are used to explain and contextualize quantitative patterns [40, 41].

The study was conducted in a 15-bed postoperative ward at a Swedish university hospital where TENS devices were readily available. Although a written protocol for initiating TENS treatment existed, it was not routinely applied in everyday practice. TENS administration did not require a formal physician’s prescription, allowing nurses to initiate treatment autonomously guided by clinical judgment and existing explicit criteria and guidelines.

The first intervention (X1) comprised a 45-minute interactive workshop delivered to all HCPs on the ward by the first author. The session combined theoretical content on the evidence base for TENS and the underlying mechanisms of action with structured, hands-on training. Participants practiced patient assessment, reviewed indications and contraindications, and received step-by-step guidance on device handling, electrode placement, program selection, and current adjustment. Practical exercises addressed common challenges such as inadequate stimulation, electrode wear, and troubleshooting of technical issues. The workshop concluded with participants testing the device on themselves and receiving a stepwise guide to support independent practice, with the aim of strengthening both competence and confidence in routine use. The instructions provided to participants were based on a standardized clinical protocol for initiating TENS in postoperative pain management from the department where the study was conducted. No specific patient subgroup was targeted, and TENS was not mandated as the first-line option but was introduced as an evidence-based and readily available alternative within multimodal pain management. Furthermore, TENS devices were already available on the ward for clinical practice. Four weeks after the workshop, a follow-up measurement (O2) was conducted to evaluate changes in TENS use.

The second intervention (X2) was a 12-week nudging phase aimed at increasing the salience of TENS as a treatment option. This stage was intentionally designed as an asynchronous and a passive strategy that required no active engagement or specific timing from the HCPs [8, 13], thereby minimizing workflow disruption. The intervention used visual prompts consisting of laminated A4 paper posters, measuring 21.0 cm in width and 29.7 cm in height, with the question *“Have you offered TENS today?”* accompanied by images of TENS devices in varying sizes and formats (some of the A4 posters were cut into smaller formats). To enhance contextual relevance and acceptability, the design and placement of materials were co-developed with two ward-based HCPs. The first and last author collaborated to review and adjust both content and positioning biweekly, ensuring novelty and visibility. Posters were strategically placed in high-traffic staff areas, including workstations, restrooms, and the staff lounge, to maximize repeated exposure and integration into daily routines. This nudging phase was intended to build on the initial educational workshop, reinforcing knowledge and confidence gained during X1 while prompting translation into consistent clinical action over time. At the conclusion of the nudging phase, a second follow-up measurement (O3) was conducted to evaluate changes in TENS use.

To ensure procedural transparency, the study was introduced at an informational meeting where the design, objectives, and roles of the research team were outlined. All participating HCPs were informed in advance that visual prompts promoting TENS use would be displayed within their clinical environment. During the 12-week nudging phase, the research team maintained a low level of presence on the ward. HCPs were informed that additional training or support could be requested at any time, although no such requests were made. The only active involvement from the research team consisted of biweekly adjustments of poster content and placement, as described above.

### Participants and data collection

In line with the explanatory sequential mixed methods design [40, 41], quantitative data on TENS use were collected first, followed by qualitative interviews. The study was conducted from January to May 2025. Quantitative data were collected prospectively during three consecutive study phases: a 5-week baseline observation phase before the educational intervention, a 4-week post-education follow-up phase after the educational workshop, and a 12-week nudging phase following initiation of the visual prompts.

Quantitative data were collected through a shared logbook maintained by all HCPs at the postoperative ward. All HCPs who administered TENS were instructed to record each treatment per unique patient, rather than repeated sessions with the same patient, and to continuously note the date and the patient’s surgical specialty (gynecology or urology). The recording was anonymous, neither patients nor HCPs could be identified. These records were used to track changes in documented TENS use across the study phases. Denominator data, including average weekly census, total postoperative patient volume, the number of patients eligible for TENS, and phase-specific case-mix data, were not available due to organizational data access constraints during the study period. Therefore, the quantitative analysis was limited to recorded TENS administrations per unique patient and the proportion of study weeks with at least one recorded TENS administration.

Qualitative data collection took place in May 2025, immediately after completion of the 12-week nudging phase. Semi-structured interviews were conducted with eligible HCPs to contextualize and explain the observed usage patterns. Written informed consent was obtained prior to each interview, with verbal reconfirmation provided before audio recording.

For the interviews, a purposive, total-population sampling strategy was employed, targeting all eligible HCPs at the study site. Recruitment was led by the first author through direct contact during site visits, supplemented with follow-up emails. HCPs who had worked at least ten shifts during the nudging phase were invited to participate in interviews (*n* = 17), of whom thirteen consented. As the final sample represented the majority of those exposed to the intervention, the study was deemed to have sufficient information power to address its qualitative aims [42].

All interviews were conducted in Swedish using the digital videoconferencing platform Microsoft Teams by the first author, who had no prior relationship with participants, using a semi-structured guide (Supplementary File 1). Sessions were audio-recorded, lasted a median of 53 min (range 45–60 min), and were transcribed verbatim. The first author checked the transcripts for accuracy against the audio recordings. The audio recordings were deleted after transcription and verification. All data were pseudonymized and stored in accordance with university data protection guidelines [43]. Quotes were translated into English and professionally edited (Editage Ltd.), with back-translation by the research team to ensure fidelity to the original meaning.

### Analysis

Descriptive statistics were used to calculate the frequency and proportion of patients receiving TENS across the three phases (O1, O2, O3). Differences in weekly TENS use between phases were assessed through pairwise comparisons with Bonferroni-adjusted significance levels. Differences in the proportion of weeks with any TENS use were examined using Fisher’s exact test. All quantitative analyses were conducted in IBM SPSS Statistics (version 29.0.2.0 (20)), with significance set at *p* < .05. Given the single-group pre–post design and the absence of denominator data, the quantitative analyses were intended to describe changes in recorded TENS administrations across study phases rather than to estimate causal effects. Weekly recorded TENS administrations and the proportion of weeks with any recorded TENS use were selected as pragmatic implementation indicators because patient-level eligibility and total postoperative patient volume were not available. Pairwise comparisons between phases were therefore interpreted as exploratory comparisons of documented use over time. Bonferroni adjustment was applied to reduce the risk of type I error across multiple phase comparisons.

Qualitative data were thematically analyzed using a deductive approach guided by the six-phase framework described by Braun and Clarke [44]. The first author familiarized himself with the data by reading and re-reading the transcripts and comparing them with the audio recordings during transcription checking. An initial deductive coding framework was developed based on the study aim and predefined areas of interest, including facilitators, barriers, responses to the educational workshop, responses to the nudging strategy, and perceived changes in clinical reasoning and practice. The first author then coded all transcripts in NVivo (QSR International, Version 14), allowing additional subcodes to be added when relevant to capture nuances within the predefined areas.

Codes were subsequently reviewed, grouped, and refined into broader thematic domains that explained and contextualized the quantitative patterns of TENS use across the study phases. To enhance analytic credibility, key codes, candidate themes, interpretations, and illustrative quotations were discussed with the last author throughout the analytic process. Disagreements or uncertainties were resolved through discussion and by returning to the transcripts. The final thematic domains were reviewed against the coded data and the full dataset to ensure that they adequately reflected participants accounts and were relevant to the explanatory mixed methods design.

### Mixed methods integration

Integration of quantitative and qualitative findings was conducted at the interpretation and reporting stages. Quantitative data on documented TENS use were first analyzed to identify patterns across the baseline, post-education, and post-nudging phases. Qualitative interview data were then used to explain and contextualize these patterns, with particular attention to how HCPs perceived the educational workshop, the visual prompts, and the integration of TENS into everyday clinical reasoning.

The findings from both data sources were compared, interpreted, and synthesized to provide a more contextualized understanding of documented practice change. A joint display was developed to make this integration explicit by linking quantitative results with qualitative explanations and integrated interpretations in accordance with the explanatory sequential design [41].

## Results

A total of 13 of 17 HCPs participated in the interviews (Table 1). The sample was predominantly female and mainly comprised nurses with specialties in anesthesia, intensive care, emergency medicine, or combined anesthesia and critical care, alongside two anesthesiologists. Participants reported long professional experience (median 20 years), but shorter tenure at the current clinic (median 3.5 years). Several held formal leadership roles, including section leaders, a ward manager, and a senior consultant physician. Participants reported limited prior experience with TENS. A few recalled earlier administrations for postoperative nausea, while others described personal experiences of receiving TENS, for example during childbirth or for back pain. These experiences ranged from positive to mixed. Despite this limited exposure, participants consistently expressed favorable attitudes toward TENS as a low-risk, non-pharmacological option that could complement existing pain treatment strategies.

**Table 1:** Participant characteristics

Characteristic	*n*	%	Median (Range)
Gender			
Female	10	77	
Male	3	23	
**Profession and specialty**			
Nurses – Anesthesia	7	54	
Nurses – Intensive care	1	8	
Nurses – Emergency medicine	1	8	
Nurses – Combined anesthesia and critical care	2	15	
Physicians – Anesthesiologist	2	15	
**Age**,** years**			46 (32–64)
**Professional experience**,** years**			20 (9–40)
**Years at current department**			3.5 (2–30)
**Leadership roles**			
Operations managers	2	15	
Department head	1	8	
Senior consultant physician	1	8	

Values are presented as n, (%), or median (range)

The results are presented by first reporting quantitative patterns of documented TENS use across the baseline, education, and nudging phases, followed by qualitative findings that explain and contextualize these patterns. The integrated presentation is organized around four explanatory domains: (1) continued but variable documented TENS use, (2) integrating TENS into postoperative pain care, (3) clinical judgment and patient-reported outcomes, and (4) early routinization and the limits of nudging (Table 2).

**Table 2:** Joint display linking quantitative results, qualitative explanations, and integrated interpretations

Integrated explanatory domain	Quantitative results	Qualitative explanations	Integrated interpretation
Sustained but variable adoption of TENS	Recorded weekly TENS use increased significantly during both the education phase (*p* = .018) and the nudging phase (*p* = .045) compared with baseline. No significant difference was observed between the education and nudging phases.	Participants described the nudging strategy as a subtle but persistent reminder, for example “like a small light in the background” (P3), that kept TENS visible in everyday practice. However, engagement varied over time, and some participants reported that the prompts gradually became less noticeable.	The quantitative increase in TENS use indicates that the education and nudging interventions supported adoption compared with baseline. The qualitative findings explain how education initiated use, while nudging helped sustain attention over time. However, the reduced salience of visual prompts also highlights the limits of passive nudging as a stand-alone sustainment strategy.
Integrating TENS into postoperative pain care	TENS was administered in 100% of weeks during the education phase and 75% of weeks during the nudging phase, compared with 0% of weeks during baseline.	HCPs described TENS as becoming part of their clinical repertoire, for example as “an extra tool” (P4). They also valued TENS as a non-pharmacological option that could help reduce reliance on medication in selected patients.	The quantitative pattern shows a shift from no recorded use at baseline to repeated use during both intervention phases. The qualitative findings expand this by showing that TENS was not only used more often but was also reframed as a legitimate and accessible component of multimodal postoperative pain care.
Clinical judgment and patient-reported outcomes	Of the recorded TENS administrations, most were provided to gynecology patients (81%), while fewer were provided to urology patients (19%).	Participants described TENS use as guided by clinical judgment, patient characteristics, and perceived patient response. Positive feedback, such as patients appearing satisfied or comfortable, reinforced continued use. In contrast, practical barriers, including surgical dressings and electrode placement, were described as limiting use in some urology patients.	The quantitative distribution across surgical specialties is explained by qualitative accounts of patient selection and practical feasibility. Together, the findings suggest that TENS adoption was not uniform across patient groups but was shaped by clinicians’ assessment of suitability, expected benefit, and practical constraints.
Early routinization and limits of nudging	TENS use was sustained during most weeks of the nudging phase, although weekly administrations varied.	Some participants described TENS as becoming part of their routine practice and expressed an intention to continue using it, for example “I’ll keep using it” (P9). Others described declining engagement over time as the prompts blended into the clinical environment.	The sustained but variable use during the nudging phase suggests early routinization for some HCPs. However, the qualitative findings also indicate that passive visual prompts may lose impact over time. Long-term sustainability may therefore require additional organizational reinforcement, such as refreshed prompts, feedback or local super users.

TENS = transcutaneous electrical nerve stimulation; HCPs = healthcare professionals; P = participant.

### Sustained but Variable Adoption of TENS

**Fig. 1:**
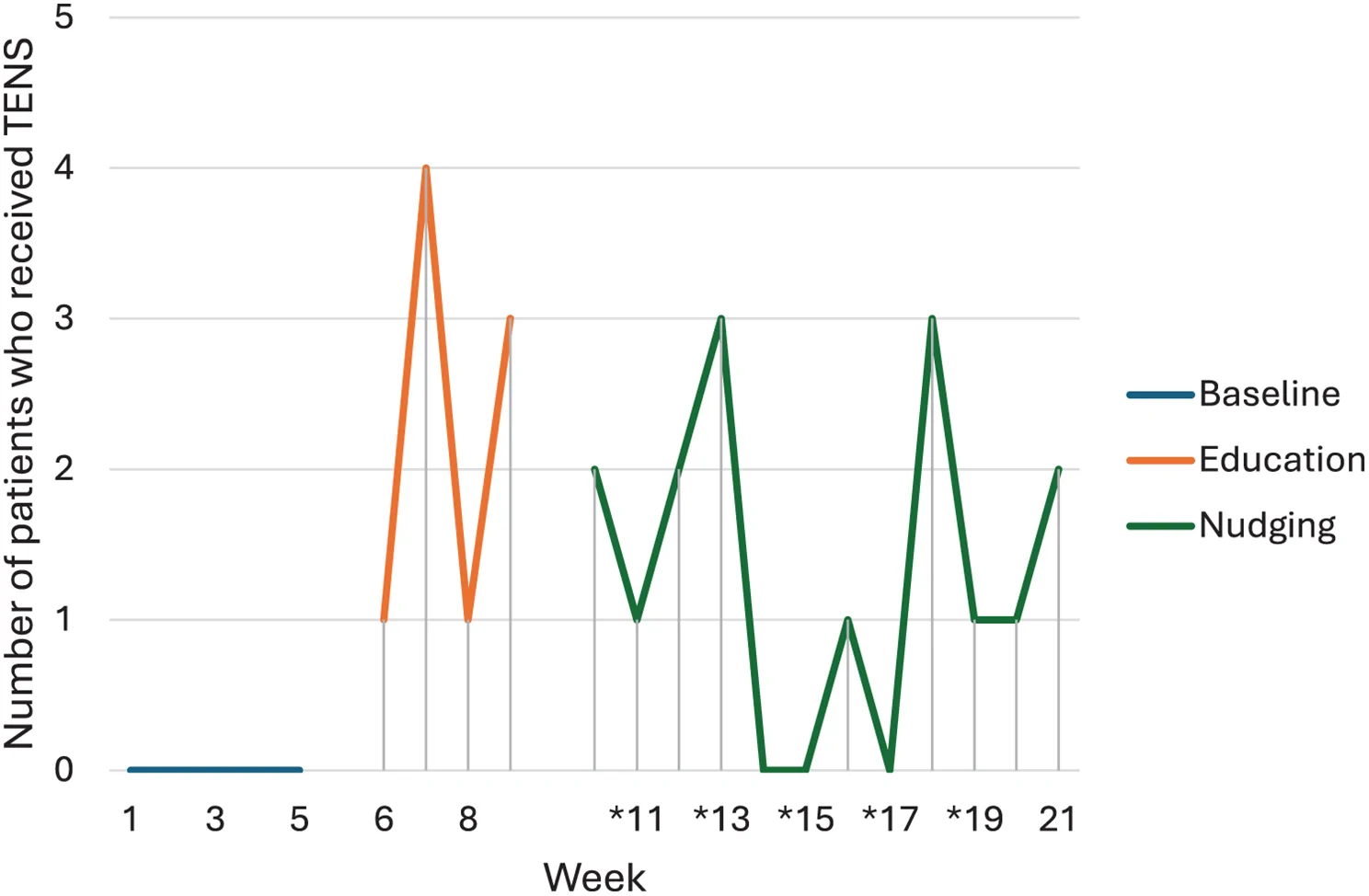
Weekly TENS administrations across the baseline, education, and nudging phases. Asterisks (*) denote weeks in which adjustments were made to the nudging materials. TENS = transcutaneous electrical nerve stimulation

The number of patients receiving TENS varied across the three study phases (Fig. 1). During the baseline phase (weeks 1–5), no patients were treated with TENS. Following the educational intervention (weeks 6–9), usage increased, peaking at four unique patients in week 7. During the nudging phase (weeks 10–21), TENS was administered in most weeks, though with greater fluctuation, ranging from zero to three unique patients per week. Pairwise comparisons of weekly TENS administrations showed significantly higher frequencies during both the education phase (Phase 2) and the nudging phase (Phase 3) compared with baseline (Phase 1) (adjusted *p* = .018 and 0.045, respectively; Table 3). No significant difference was observed between the two intervention phases (Phase 2 vs. Phase 3, adjusted *p* = 1.000).

**Table 3:** Bonferroni-adjusted pairwise comparisons of weekly TENS administrations between study phases

Comparison (Phases)	Test Statistic	Std. Error	Std. Test Statistic	Adj. Sig.
Phase 1 (Baseline) – Phase 3 (Nudging)	-7.708	3.174	-2.429	0.045
Phase 1 (Baseline) – Phase 2 (Education)	-11.000	4.000	-2.750	0.018
Phase 3 (Nudging) – Phase 2 (Education)	3.292	3.442	0.956	1.000

TENS = transcutaneous electrical nerve stimulation; Std. Error = standard error; Std. Test Statistic = standardized test statistic; Adj. Sig. = Bonferroni-adjusted significance.

 This week-to-week variation was reflected in the interviews. Some participants reported frequent use (“I’ve used it maybe 3–4 times a week,” P9), while others described a slower uptake (“zero to five or six times,” P4). For some, the nudging served as a subtle but persistent reminder (“like a small light in the background”, P3) that kept TENS in mind and encouraged trial use. Visual prompts also stimulated collegial discussions (“I’ve heard many more discussions about TENS now than before,” P10), reinforcing collective awareness. However, several participants noted that their use declined over time as the prompts lost salience (“interest declined,” P11) or became part of the background environment.

### Integrating TENS into Postoperative Pain Care

In addition to increases in total weekly TENS administrations, the proportion of weeks with at least one recorded use differed significantly across phases (*p* = .002). As shown in Table 4, no administrations were recorded during the baseline phase (0/4 weeks), TENS was administered in every week of the education phase (4/4 weeks), and in three-quarters of the nudging phase (9/12 weeks).

**Table 4:** Proportion of study weeks in which TENS was administered across the three study phases (Baseline, Education, Nudging)

Phase	Weeks with TENS (*n*)	Total weeks (*n*)	Proportion (%)
Baseline	0	5	0%
Education	4	4	100%
Nudging	9	12	75%

TENS = transcutaneous electrical nerve stimulation; n = number of weeks

Qualitative findings indicated that this shift was accompanied by a reframing of TENS within participants clinical reasoning. Several participants described an expanded sense of treatment capacity, for example by referring to TENS as “an extra tool” (P4), and described moving from viewing TENS as a peripheral option to recognizing it as a viable component of multimodal postoperative pain care. Education and nudging appeared to make TENS more accessible in everyday practice and strengthened openness toward non-pharmacological strategies, as reflected in the statement: “It made me think that you don’t always have to go straight to injections” (P6). Some participants linked this change to patient-centered considerations, such as keeping down medication use, while visual prompts and collegial dialogue further normalized TENS use and fostered a shared sense of legitimacy in offering it as part of routine care.

### Clinical judgment and patient-reported outcomes

Among the 26 recorded TENS administrations, 21 (81%) were provided to gynecology patients and 5 (19%) to urology patients. Interview data suggested that administration decisions were guided by clinical judgment and situational factors, with HCPs frequently citing specific patient characteristics as influential (“They shouldn’t have too high/low VAS scores,” P5; “I’ve been able to keep down the medications,” P9). Positive patient feedback further supported continued use (“They looked satisfied… content,” P8). Overall, TENS was regarded as most beneficial in patients who had underwent gynecology surgery, where HCPs described it as particularly helpful for managing postoperative pain after gynecological procedures. In contrast, its use in urology was perceived as more limited, with several HCPs highlighting practical barriers such as the placement of surgical dressings, which complicated assessment of appropriate electrode positioning. At the same time, some described isolated positive cases (*“*I have only used it once for urgency, but it worked very well…. When you see that it works, you are also motivated to use it more.*“*, P4).

### Early routinization and limits of nudging

Although TENS administration declined after the education phase, TENS was still applied in most weeks during the nudging period (75%). For some, repeated use led to routinization (“Once you’ve done it a few times, it becomes part of your practice,” P4) and an expressed intent to continue (“I’ll keep using it,” P9). Others described a lasting shift in clinical reasoning, with TENS now “there in the back of my mind” (P3). However, several participants reported reduced engagement over time (“Only in the beginning,” P7; “I haven’t used it myself recently,” P10). Contributing factors included the visual prompts becoming less salient (“blending into the environment,” P12).

## Discussion

This study examined a brief educational session followed by 12 weeks of visual prompts embedded in the clinical environment to support TENS use in acute postoperative pain management. The results suggest that even modest, low-resource strategies might influence documented practice patterns, reframe perceptions of a non-pharmacological modality, and stimulate dialogue in the postoperative context.

However, the findings should be interpreted cautiously. Because denominator data were unavailable and the study used a single-group pre–post design without a control group, the observed changes should be understood as changes in documented TENS use over time rather than as rates of adoption among eligible patients or causal evidence of an independent nudging effect. Nevertheless, the absence of recorded TENS administrations during baseline, despite available devices and a local protocol, suggests that TENS was not documented as part of routine practice before the interventions.

While facilitation is typically conceptualized as an active process enacted by individuals who align the innovation, recipients, and context [45], our findings extend this concept to include nudging. In this form, asynchronous and passive nudges function as point-of-care environmental cues that facilitate the implementation of an intervention while preserving professional autonomy. In our study, the nudging phase exemplified this approach. Prompts required no active engagement at specific times [8, 13], yet appeared to keep TENS visible as a possible option in clinical reasoning. For some participants, repeated exposure to these cues contributed to routinization and confidence in offering TENS, while for others, it triggered collegial conversations that reinforced its legitimacy within multimodal pain management.

The educational component addressed previously reported barriers, such as lack of limited practical experience with TENS [36], and provided foundational knowledge and skills, which were then reinforced through the nudging phase. Although the weekly frequency of TENS use fluctuated, usage remained higher than baseline throughout the intervention period. This suggests that passive and asynchronous nudging might have contributed to continued documented use over time, even when the initial educational input is brief. Previous research suggests that educational meetings and guideline dissemination can support professional practice, but their effects on clinical behavior are often modest, variable, and context dependent when used alone [2, 3]. In this study, the visual prompts might have complemented the educational workshop by keeping TENS visible in the clinical environment after the initial training.

At the same time, several participants reported that prompts became less noticeable over time, echoing findings on stimulus habituation [46]. Without renewal or variation, the capacity of visual cues to sustain change may diminish. This aligns with the broader literature suggesting that passive strategies are most effective when periodically refreshed or combined with intermittent active facilitation [47, 48]. In this study, such variation was implemented through biweekly changes in placement and form. However, to ensure long-term sustainability and prevent gradual decline, future research could explore similar adaptive designs paired with additional nudge components, such as providing feedback on practice patterns, to sustain attention and motivation [7], or non-nudge components such as providing clear and context-specific clinical guidelines, and designating formal local super users (i.e., HCPs with specific competence and confidence in using TENS, who actively integrate it into their own practice and serve as practical and motivational resources for colleagues). Local champions or super users may support continued adoption by providing peer-based guidance and practical support, particularly when they have a clear mandate, dedicated time, and adequate training [49, 50]. Such measures can help institutionalize TENS use, support peer-to-peer learning, and ensure that the behavioral gains achieved through education and nudging are maintained over time.

Our findings also highlight the role of clinical judgment in determining when and for whom TENS is offered. The educational session did not specify patient groups. This left room for context-specific reasoning, with clinicians frequently favoring gynecology patients, likely because electrode placement was perceived as easier in this group. In contrast, in urology, surgical dressings were often described as obstructing access and complicating assessment of appropriate electrode positioning, which may have constrained adoption. Positive patient feedback reinforced continued use, whereas perceived limitations in certain patient groups constrained adoption. In this way, the facilitation provided by visual prompts helped keep TENS within HCPs field of attention, but decisions to administer it were still shaped by their appraisal of patient suitability. This reflects the principles of nudge theory, where decision-making environments are designed to facilitate behavior change without restricting autonomy [4]. Although such passive nudges can operate “under the radar” and influence decisions without explicit reflection [7], their interaction with professional judgment in this study illustrates how facilitation, through passive and asynchronous nudges can normalize a practice while both respecting and relying on clinical autonomy. Further, the transparent nature of the nudge approach, where participating HCPs were explicitly informed that visual prompts promoting TENS would be introduced into their clinical environment, may have reinforced perceptions of professional autonomy. This aligns with evidence suggesting that transparency does not inherently reduce the effectiveness or acceptance of nudges, except among individuals who view them as a threat to their autonomy [51].

This low-cost, low-disruption educational and nudge strategy may have a meaningful clinical impact, as systematic reviews show that nudge interventions commonly produce statistically significant improvements in clinician behavior with effect sizes comparable to more resource-intensive methods [52, 53]. In this case, TENS administration was supported by local guidelines yet had no recorded use during the baseline phase prior to the intervention. The educational component consisted of a single, brief 45-minute session, after which the nudging strategy maintained TENS practice beyond the education phase. While nudging has been shown to be cost-effective in prior research, formal economic evaluations remain limited, with only about one-third of studies including such analyses [54], underscoring the need for more comprehensive assessments of long-term value. In this study, even with minimal financial and logistical resources, the combined strategy influenced clinical behavior in ways that could improve patient outcomes. A simplified cost illustration was developed to provide context for the modest resources required by this intervention (Table 5).

Statistics Sweden (SCB) reported that the average monthly salary for an anesthesia specialist nurses in 2024 was approximately USD 4,630 [55]. Assuming 174 working hours per month, this corresponds to approximately USD 27 per hour. In Scenario A, costs were limited to staff time for a single 45-minute educational session attended by 13 participants and two educators. This equaled approximately 14 staff hours in total (15 × 0.75 h), corresponding to approximately USD 367. Scenario B added five hours for designing the visual prompts, corresponding to approximately USD 132 and resulting in a total of approximately USD 498. Scenario C further included the purchase of five new TENS devices (approximately USD 609) and 50 electrode packs (approximately USD 465). The combined additional equipment cost was approximately USD 1,074, yielding a total of approximately USD 1,570. Because TENS equipment was already available in the ward, this scenario represents a conservative overestimation. Printing and lamination were excluded because these were covered by existing ward resources.

**Table 5:** Simplified cost estimation of the intervention

Scenario	Description	Estimated cost (USD)
A	45-min workshop for 13 participants + 2 educators (staff time only)	367
B	Scenario A + 5 h design time for visual prompts	498
C	Scenario B + 5 TENS devices + 50 packs electrodes	1,570

TENS = transcutaneous electrical nerve stimulation; USD = United States dollars; h = hours.

Even when accounting for new equipment, the estimated costs of the intervention remained modest. This estimate should be interpreted as a simplified cost illustration rather than a formal economic evaluation. Implementation science literature emphasizes that cost data are important for assessing feasibility and scalability but are often inconsistently reported and highly context dependent [56, 57]. In this context, the present cost illustration suggests that a brief educational session combined with low-cost visual prompts may be feasible to deliver with limited financial and logistical resources. Future studies should include formal economic evaluations to assess long-term value, scalability, and cost-effectiveness across different clinical settings.

Importantly, the strategy facilitated TENS practice and supported multimodal pain management. Participants reported that increased TENS administrations, particularly in appropriately selected patients, reduced the need for systemic analgesics, including opioids, consistent with prior evidence showing that TENS is a low-risk, cost-effective modality capable of reducing pharmacological requirements [28–32]. Because the strategy is simple to produce, relies only on laminated A4 posters, and can be implemented by local HCPs, it is highly scalable. While TENS devices may not be available in all postoperative units, they can be procured at relatively low cost, which further supports the feasibility of wider implementation. Similar complementary nudge-based approaches could be adapted to promote the adoption and sustainment of other underutilized, evidence-based interventions across a variety of clinical contexts.

### Limitations

This study has several limitations. As a single-site, quasi-experimental study without randomization or a control group, the design limits causal inference and generalizability. However, this design was considered appropriate for the pragmatic aim of exploring implementation in a real-world clinical context [58]. The observed changes in documented TENS use cannot be attributed to the nudging phase alone, as the independent contribution of nudging cannot be distinguished from the preceding educational workshop, temporal trends, patient-related factors, or other contextual influences. In addition, because no significant difference was observed between the education and nudging phases, claims regarding sustainment should be interpreted cautiously. The findings indicate continued documented TENS use during the nudging phase, but do not establish that nudging independently sustained adoption.

The quantitative findings should be interpreted considering the relatively small number of recorded TENS administrations (*n* = 26) and the absence of denominator data. Due to organizational constraints, average weekly census data, total postoperative patient volume, the number of patients eligible for TENS, and phase-specific case-mix data were not available. Therefore, it was not possible to calculate TENS use rates or determine whether changes in recorded administrations reflected increased adoption, variation in patient volume, differences in case mix, patient eligibility, or a combination of these factors. The absence of recorded TENS administrations during baseline should therefore be understood as no documented baseline use rather than as a denominator-based estimate of baseline adoption. Future studies should include denominator data and patient-level eligibility criteria to enable more precise assessment of adoption.

Data collection relied on self-reported accounts, including logbook entries and interviews, which may be subject to incomplete reporting, recall bias, or social desirability bias. Compliance with logbook reporting could not be independently verified, and some TENS administrations may therefore have been undocumented. Although integration of quantitative and qualitative data can strengthen interpretation by combining measurable patterns with contextual explanations [40, 41], the findings should be understood as reflecting documented practice rather than complete patient-level implementation data.

Although the interview sample represented the majority of eligible HCPs exposed to the intervention and was considered to provide sufficient information power [42], the sample was relatively small and limited to one clinical setting. In addition, coding was conducted primarily by one researcher, which may have increased the risk that coding decisions and theme development were influenced by the first author’s pre-understanding and analytic perspective. To mitigate this risk, key interpretations, candidate themes, and illustrative quotations were discussed with the last author, and qualitative findings were integrated with quantitative usage data. Nevertheless, future studies could strengthen analytic credibility by involving multiple coders, formal reflexive documentation, or participant validation.

Patient interviews described in the trial registration were not conducted as part of the present study due to organizational constraints. As a result, the qualitative findings reflect HCPs perspectives only and do not capture patients experiences of receiving TENS. Future research should include patient perspectives to provide a more comprehensive understanding of acceptability, perceived benefit, and barriers to TENS use from the patient standpoint.

## Conclusion

A brief, single-session educational workshop followed by an asynchronous and passive nudging strategy was associated with increased and continued documented use of TENS for postoperative pain managment during the study period. The nudging phase maintained attention to TENS long after the education phase, facilitating its integration into clinical reasoning and normalizing its role as a legitimate non-pharmacological option. These findings illustrate how asynchronous, passive and transparent nudging, can complement traditional implementation strategies and facilitate practice change while respecting professional autonomy. Furthermore, this approach holds promise for supporting the adoption of other underused, evidence-based interventions across diverse clinical contexts.

## Supplementary Information

Below is the link to the electronic supplementary material.

Supplementary Material 1

## Data Availability

Data and material to reproduce the findings reported in this manuscript can be obtained from the authors upon reasonable request.
