# Supplementary material for: Brief education and nudging to facilitate TENS use in postoperative care: a quasi-experimental mixed methods study
Source: BMC Health Serv Res. 2026 Oct 27;26:1479. doi: 10.1186/s12913-026-15913-0 (PMC13715202; doi:10.1186/s12913-026-15913-0)
Supplement: Supplementary file 1 [file 12913_2026_15913_MOESM1_ESM.docx]

**Supplementary File 1. Semi-structured interview guide**

The interview guide began with the following open-ended question:

***Can you describe what it was like for you to work during the period when the TENS posters were displayed in the clinical environment?***

Follow-up prompts were used flexibly to deepen the understanding of specific dimensions such as the interventions perceived impact on practice.

**Example follow-up questions:**

1. How did you experience the educational workshop?
2. How would you describe your experience of the nudging elements?
3. What influenced your decision to use TENS?
   - or not to use TENS?
4. Is there anything you would have wished to be different regarding the nudging?
   - or the educational intervention?

**Demographic questions:**

6. Age
7. Sex
8. Professional role (e.g., nurse, anesthesia nurse, physician)
9. Years of clinical experience
10. Years working at the current postoperative ward
